# A review of bottom-up vs. top-down control of sponges on Caribbean fore-reefs: what’s old, what’s new, and future directions

**DOI:** 10.7717/peerj.4343

**Published:** 2018-01-31

**Authors:** Joseph R. Pawlik, Tse-Lynn Loh, Steven E. McMurray

**Affiliations:** 1 Department of Biology and Marine Biology and Center for Marine Science, UNCW, Wilmington, NC, USA; 2 Quest University Canada, Squamish, BC, Canada

**Keywords:** Coral reefs, Ecology, Food limitation, Predation, DOC DOM, Sponge-loop, Vicious circle, Hawksbill turtles, Historical ecology, Ecosystem function

## Abstract

Interest in the ecology of sponges on coral reefs has grown in recent years with mounting evidence that sponges are becoming dominant members of reef communities, particularly in the Caribbean. New estimates of water column processing by sponge pumping activities combined with discoveries related to carbon and nutrient cycling have led to novel hypotheses about the role of sponges in reef ecosystem function. Among these developments, a debate has emerged about the relative effects of bottom-up (food availability) and top-down (predation) control on the community of sponges on Caribbean fore-reefs. In this review, we evaluate the impact of the latest findings on the debate, as well as provide new insights based on older citations. Recent studies that employed different research methods have demonstrated that dissolved organic carbon (DOC) and detritus are the principal sources of food for a growing list of sponge species, challenging the idea that the relative availability of living picoplankton is the sole proxy for sponge growth or abundance. New reports have confirmed earlier findings that reef macroalgae release labile DOC available for sponge nutrition. Evidence for top-down control of sponge community structure by fish predation is further supported by gut content studies and historical population estimates of hawksbill turtles, which likely had a much greater impact on relative sponge abundances on Caribbean reefs of the past. Implicit to investigations designed to address the bottom-up vs. top-down debate are appropriate studies of Caribbean fore-reef environments, where benthic communities are relatively homogeneous and terrestrial influences and abiotic effects are minimized. One recent study designed to test both aspects of the debate did so using experiments conducted entirely in shallow lagoonal habitats dominated by mangroves and seagrass beds. The top-down results from this study are reinterpreted as supporting past research demonstrating predator preferences for sponge species that are abundant in these lagoonal habitats, but grazed away in fore-reef habitats. We conclude that sponge communities on Caribbean fore-reefs of the past and present are largely structured by predation, and offer new directions for research, such as determining the environmental conditions under which sponges may be food-limited (e.g., deep sea, lagoonal habitats) and monitoring changes in sponge community structure as populations of hawksbill turtles rebound.

## Introduction

These are exciting times for researchers in the area of sponge ecology. Mostly unnoticed in past studies of Caribbean reefs, sponges are gaining considerable attention as independent reports show that sponge cover has increased dramatically in the wake of ongoing losses of coral cover (Norstrom et al., 2009; Villamizar et al., 2013; de Bakker et al., 2017). On Florida’s reef track, long-term studies reveal a 122% increase in the density of the giant barrel sponge (*Xestospongia muta*), mirroring abundance gains across its range, with this species now likely the dominant organism on Caribbean reefs in terms of biomass (McMurray, Finelli & Pawlik, 2015). Multiple research groups have confirmed that diverse sponge species eat dissolved organic carbon (DOC) in addition to particulate organic carbon (POC), but that DOC constitutes the largest part of the diet of these species (McMurray et al., 2016; Rix et al., 2016). Additionally, some sponges rely on autotrophic microbial symbionts for part of their nutrition (Erwin & Thacker, 2008; Freeman et al., 2015), with the range of abundance and dependence on symbionts across sponge species an area of great research interest (Poppell et al., 2013; Gloeckner et al., 2014). And because these microbial assemblages may include taxa that transform nutrient molecules (Southwell et al., 2008; Morganti et al., 2017), sponges may be sources of fertilizer that promote the growth of seaweeds on reefs (Pawlik, Burkepile & Thurber, 2016). Sponges can process large volumes of seawater with their pumping and filtering activities (Reiswig, 1974); hence, their influence can reach well into the pelagic realm, with estimates that giant barrel sponges can overturn the water column every 2.8–6 days (McMurray, Pawlik & Finelli, 2014). In various combinations, these developments have prompted hypotheses that greatly alter our understanding of ecosystem function on coral reefs, including the “sponge-loop” that proposes recycling of coral and seaweed-produced DOC by sponges as sloughed cellular debris that returns carbon to the benthos (de Goeij et al., 2013; de Goeij, Lesser & Pawlik, 2017), and the “vicious circle” that provides an explanation for the lack of resilience of Caribbean coral reefs based on a positive feedback loop of DOC and nutrients between seaweeds and sponges that enhances the growth of both to the detriment of corals (Pawlik, Burkepile & Thurber, 2016). Among the foregoing, there has been an interesting and lively recent exchange on the relative importance of bottom-up (food) and top-down (predation) factors that alter sponge community structure on Caribbean fore-reefs (Pawlik et al., 2015a, 2015b; Slattery & Lesser, 2015). The purpose of the present contribution is to review new citations and analyses that inform this debate, as well as older references that provide insights that were not previously addressed.

In the annals of ecology, it is likely there has been no greater disparity of opinion regarding the relative importance of bottom-up vs. top-down control of community structure than for sponges on Caribbean reefs (Pawlik et al., 2015b). Here is a very brief history:

- **No top-down control:** Randall & Hartman (1968) initially declared that sponges on Caribbean reefs were free of top-down control after enumerating the few species of reef fishes that had sponge tissue in their guts (but see further analysis below). This view was repeated by Wulff (1994), who recorded the bites that fishes took of sponges in a 16 m^2^ observation area on a Panamanian reef. While the latter study did not document the tissue volume that fish actually consumed (fishes may bite at objects without eating them), the author concluded that sponge-eating fishes actively alternate feeding on various sponge species (smorgasbord or rotational feeding) to avoid sponge defenses, and therefore have no particular impact on any one sponge species. These conclusions were reported without reference to bottom-up processes.
- **Yes, top-down control:** Using laboratory and field manipulative experiments, it was demonstrated that sponge-eating fishes have clear preferences among sponge species, particularly based on chemical defenses (Pawlik et al., 1995; Pawlik, 1997, 1998; Hill, 1998; Hill & Hill, 2002), that parrotfishes were important spongivores (Dunlap & Pawlik, 1996, 1998), and that top-down effects were important (Pawlik, 2011). These conclusions were reported without reference to bottom-up processes.
- **Only bottom-up control, no top-down control:** Citing Randall & Hartman (1968) for the absence of top-down control on Caribbean sponges, and based on studies of the relationship between the concentration of living particulate food in seawater as a function of depth and sponge abundance, size and tube elongation, Lesser and colleagues asserted that sponge community structure was unusual in being principally driven by bottom-up effects (Lesser, 2006; Trussell et al., 2006).
- **Primarily top-down control, no evidence for bottom-up control:** Pawlik et al. (2013) conducted manipulative sponge growth experiments employing whole-sponge wet mass as the response variable on the same reef as Trussell et al. (2006), but also excluded sponge predators as part of the experimental design, and found strong evidence for an effect of predation on sponge growth, but not for food-limitation. Lesser & Slattery (2013) responded with additional correlative evidence linking depth-dependent picoplankton availability with sponge biomass and tube elongation. Pawlik et al. (2015a) countered by surveying the literature, and found no evidence for food limitation of Caribbean reef sponges when only particulate food was considered, noting that Lesser and colleagues had not considered DOC as a potential source of food for sponges. Citing the diverse and abundant potential sources of food available on fore-reefs to sponges, including DOC, Pawlik et al. (2015b) proposed that sponge community structure on Caribbean reefs was driven primarily by top-down effects.

## Survey Methodology

Since 2013, and the publication of the sponge-loop paper (de Goeij et al., 2013) as well as the first exchange over the importance of food-limitation vs. predation (Lesser & Slattery, 2013; Pawlik et al., 2013), there has been a surge of new publications on sponge feeding biology and ecology. In the sections below, we discuss these new developments relative to the bottom-up and top-down debate, add some insights from older publications that were not previously considered, and provide new research directions. For new studies, we used standard search methods (e.g., Web of Science, Google Scholar) to identify over 10 papers since 2015 that made new discoveries specifically related to sponge feeding alone, along with many more citations that have an important bearing on the broader topics relevant to this review.

## Bottom-up Control—What’s New?

The contention that variability in the structure of sponge communities on Caribbean fore-reefs is principally explained by bottom-up processes was thoroughly reviewed in Pawlik et al. (2015a), with follow-up responses by Slattery & Lesser (2015) and Pawlik et al. (2015b). In brief, bottom-up control is primarily founded on correlative evidence relating sponge distributions, abundances and rates of sponge tube elongation to the concentration of sponge food as living particulate organic carbon (LPOC) as picoplankton in the water column. The arguments against bottom-up control included the absence of evidence for food limitation when considering four predicted patterns that would be expected under conditions of food limitation, a more expansive review of the literature to address those patterns, the lack of consideration of other sources of sponge nutrition, and methodological problems associated with experiments that reported evidence of bottom-up control, particularly the lack of predator-exclusion cages and inappropriate methods for measuring sponge growth (sponge tube elongation as opposed to whole sponge wet-mass determinations; Pawlik et al., 2015a). New publications that inform this debate primarily address sponge nutrition: how sponges process seawater, what sponges eat, where their food comes from, and how variable sponges are across species in their pumping activities and nutritional responses.

While sponge consumption of LPOC in the form of picoplankton has been used as a metric to infer potential food limitation, there is increasing evidence that the sponge diet is broader than initially assumed. This is significant, as LPOC represents a relatively small portion of the organic matter in seawater accessible to sponges, which is largely in the form of DOC (>90%) and detritus (Ribes, Coma & Gili, 1999b; Hansell & Carlson, 2002; McMurray et al., 2016). Since the pioneering work that suggested sponges consume DOC to satisfy their metabolic requirements (Reiswig, 1974, 1981), and the first direct evidence of sponge DOC uptake (Yahel et al., 2003), there has been an increasing number of studies demonstrating the importance of DOC for sponge nutrition. These studies have been performed by using isotopically enriched DOC as a tracer to follow sponge DOC uptake (de Goeij et al., 2008a, 2013; van Duyl et al., 2008, 2011; Rix et al., 2016, 2017) or by using direct In–Ex methods (Yahel, Marie & Genin, 2005) to quantify the concentrations of DOC in seawater before and after sponge processing (Table 1 and references therein). For studies in which both DOC and total organic carbon (TOC) were quantified, DOC frequently comprised >70% and as much as >90% of the sponge diet for the species investigated (Table 1). While uptake of DOC has long been considered limited to sponge species with a high abundance of microbial symbionts (HMA sponges; Reiswig, 1974, 1981), recent findings indicate that DOC is also an important part of the diet of sponge species with relatively lower microbial abundances (LMA sponges), suggesting that the uptake of DOC may be a common feeding strategy for sponges (de Goeij et al., 2008b, 2013; Rix et al., 2016, 2017; Morganti et al., 2017). Additionally, some sponge species appear to derive their nitrogen from microbial symbionts, while others do not (Morganti et al., 2017). Comparative studies by Hoer et al. (2018) of massive, emergent sponge species suggested that DOC uptake may be limited to HMA species on the basis of differences in DOC concentrations of incurrent and excurrent seawater samples; however, this conclusion was based on an analysis that did not consider the rate of DOC uptake (i.e., DOC flux; Table 1), which is a more relevant metric of sponge feeding, as it accounts for the generally faster pumping rates of LMA vs. HMA species (Table 1). When published data for mean pumping rates (Reiswig, 1974; Weisz, Lindquist & Martens, 2008) were used to convert DOC uptake by the sponge species examined by Hoer et al. (2018) to rates of DOC consumption, DOC flux mediated by the LMA species *Callyspongia vaginalis* was found to approach flux estimates for the HMA species *Verongula gigantea* and *X. muta*, but the LMA species *Niphates digitalis* remained a net producer of DOC and flux estimates for this species were lower than those for the HMA species investigated (Table 1). It may be that the ability of sponges to process DOC is also influenced by morphology (de Goeij, Lesser & Pawlik, 2017), as rates of DOC uptake for emergent species are, with the exception of *Ircinia strobilina*, *V. gigantea*, and *X. muta*, approximately an order of magnitude lower than those reported for encrusting species (Table 1).

**Table 1:** Sponge-mediated changes to DOC in seawater.

Sponge species	Study location	HMA/LMA	Morphology	*n*	ΔDOC (μmol C L_seawater_^−1^)	Volume flow (L s^−1^ L_sponge_^−1^)	DOC flux (μmol C s^−1^ L_sponge_^−1^)	DOC in diet (%)	Citation
*Xestospongia muta*	Florida Keys	HMA	Emergent	65	9.8 ± 13.1	–	–	–	Hoer et al. (2018)
*Xestospongia muta*	Florida Keys	HMA	Emergent	2	11.8 ± 8.5	0.045 ± 0.009	0.53 ± 0.42	96	Hoer et al. (2018)
*Ircinia strobilina*	Florida Keys	HMA	Emergent	8	26.8 ± 27.0^†^	0.093^‡^	2.49^a^	–	^†^Hoer et al. (2018), ^‡^Weisz, Lindquist & Martens (2008)
*Verongula gigantea*	Florida Keys	HMA	Emergent	6	22.7 ± 20.5^†^	0.05–0.10^‡^	1.14–2.27^a^	–	Hoer et al. (2018)^†^ and Reiswig (1974)^‡^
*Spheciospongia vesparium*	Florida Keys	HMA, LMA	Emergent	6	−1.3 ± 17.7^†^	0.176^‡^	−0.23^a^	–	Hoer et al. (2018)^†^ and Weisz, Lindquist & Martens (2008)^‡^
*Niphates digitalis*	Florida Keys	LMA	Emergent	10	−1.8 ± 5.0^†^	0.365^‡^	−0.66^a^	–	Hoer et al. (2018)^†^ and Weisz, Lindquist & Martens (2008)^‡^
*Callyspongia vaginalis*	Florida Keys	LMA	Emergent	7	2.4 ± 7.5^†^	0.374^‡^	0.90^a^	–	Hoer et al. (2018)^†^ and Weisz, Lindquist & Martens (2008)^‡^
*Mycale laxissima*	Florida Keys	LMA	Emergent	2	1.4 ± 5.9^†^	0.21–0.27^‡^	0.29–0.38^a^	–	Hoer et al. (2018)^†^ and Reiswig (1974)^‡^
*Ircinia felix*	Caribbean Sea	HMA	Emergent	18 15 18	36.7 ± 85.7 68.3 ± 82.0 1.9 ± 41.7	–	–	–	Archer et al. (2017)^b^
*Agelas oroides*	Mediterranean Sea	HMA	Encrusting	7	7.0 ± 18.5	0.312 ± 0.072^c^	2.18^a^^,^^c^	–	Morganti et al. (2017)
*Petrosia ficiformis*	Mediterranean Sea	HMA	Encrusting	6	8.0 ± 21.0	0.132 ± 0.018^c^	1.06^a^^,^^c^	–	Morganti et al. (2017)
*Chondrosia reniformis*	Mediterranean Sea	HMA	Encrusting	6	13.0 ± 19.9	0.282 ± 0.03^c^	3.67^a^^,^^c^	–	Morganti et al. (2017)
*Crambe crambe*	Mediterranean Sea	LMA	Encrusting	5	−1.0 ± 5.0	0.186 ± 0.066^c^	−0.19^a^^,^^c^	–	Morganti et al. (2017)
*Dysidea avara*	Mediterranean Sea	LMA	Encrusting	6	5.0 ± 13.0	0.426 ± 0.252^c^	2.13^a^^,^^c^	–	Morganti et al. (2017)
*Xestospongia muta*	Florida Keys	HMA	Emergent	32	10.3 ± 14.7	0.063 ± 0.003	0.65 ± 0.91	55 ± 20	McMurray, Pawlik & Finelli (2017)
*Xestospongia muta*	Florida Keys	HMA	Emergent	5	29.3 ± 23.4	0.063 ± 0.004	1.84 ± 1.42	70.2 ± 7.7	McMurray et al. (2016)
*Cliona delitrix*	Caribbean Sea	LMA	Boring	10	10.0 ± 12.0	0.008 ± 0.002^d^	0.10 ± 0.16^d^	76^e^	Mueller et al. (2014)
*Siphonodictyon* sp.	Caribbean Sea	HMA	Boring	8	13.0 ± 17.0	0.009 ± 0.003^d^	0.13 ± 0.22^d^	82^e^	Mueller et al. (2014)
*Dysidea avara*^f^	Mediterranean Sea	LMA	Encrusting	12	−0.7 ± 2.8	–	–	–	Ribes et al. (2012)
*Agelas oroides*	Mediterranean Sea	HMA	Encrusting	9	9.9 ± 4.0	–	–	–	Ribes et al. (2012)
*Chondrosia reniformis*	Mediterranean Sea	HMA	Encrusting	9	7.8 ± 3.4	–	–	–	Ribes et al. (2012)
*Halisarca caerulea*	Caribbean Sea	LMA	Encrusting	7	–	0.069 ± 0.017^g^	3.64 ± 0.69	>90^e^	de Goeij et al. (2008b)^h^
*Mycale microsigmatosa*	Caribbean Sea	LMA	Encrusting	6	–	0.068 ± 0.015^g^	4.22 ± 0.25	>90^e^	de Goeij et al. (2008b)^h^
*Merlia normani*	Caribbean Sea	LMA	Encrusting	3	–	0.051 ± 0.006^g^	3.78 ± 0.67	>90^e^	de Goeij et al. (2008b)^h^
*Theonella swinhoei*	Gulf of Aqaba	HMA	Emergent	30	6 ± 5^i^ 10 ± 8	0.043 ± 0.03	0.43 ± 0.30	40^i^ 97	Yahel et al. (2003)
*Aplysina fistularis*^j^	Caribbean Sea	HMA	Emergent	27	6.5	0.124	0.80	86	Reiswig (1981)
*Verongula reiswigi*^j^	Caribbean Sea	HMA	Emergent	11	6.1	0.085	0.52	75	Reiswig (1971) and Reiswig (1974, 1981)

**Notes:**

Sponge-mediated changes to the concentration of DOC in seawater and the relative contribution of DOC to the sponge diet for studies of demosponges in which bulk DOC was quantified. ΔDOC represents the mean ± SD change in DOC concentration between paired incurrent and excurrent seawater samples; positive values indicate net DOC consumption and negative values indicate net DOC production. Volume flow is the mean ± SD sponge pumping rate, DOC flux is the mean ± SD rate of DOC uptake (positive) or production (negative), and DOC in diet is the percentage contribution of DOC to total organic carbon (POC + DOC) consumed. DOC was defined as the organic carbon passing a 0.7 μm GF/F glass fiber filter unless otherwise noted. When indicated (†,‡), data from two studies were used to generate calculations reported herein.

a Calculated herein using reported means.

b DOC defined as the organic carbon passing through a 0.45 μm nylon-membrane filter.

c Volume flow and DOC flux estimates are standardized to surface area (cm^2^ sponge) rather than volume.

d Estimates not standardized to sponge size (i.e., volume or area). Volume flow is expressed in L s^−1^, DOC flux in μmol C s^−1^.

e TOC indirectly estimated as DOC + POC, where POC is estimated as two times the carbon contributed by bacterioplankton.

f Ribes, Coma & Gili (1999a) additionally found *Dysidea avara* to exhibit a mean ± SE production of DOC of 0.33 ± 0.15 mg C g AFDW^−1^ h^−1^.

g Clearance rate; calculated herein using data reported in de Goeij et al. (2008b).

h DOC defined as the organic carbon passing through a 0.2 μm polycarbonate filter.

i Excurrent DOC conservatively estimated from measurements of TOC with the assumption that all incurrent POC was consumed.

j DOC uptake estimated on the basis of the sponge carbon budget.

A consistent finding that has emerged from the growing body of work on sponge feeding that further complicates between-species comparisons of DOC consumption is the high intraspecific variability in DOC uptake (Table 1). For example, mean rates of DOC consumption for the sponge *Ircinia felix* at three Caribbean sites ranged from 1.94 to 68.27 μmol C L^−1^ and the standard deviation of estimates surpassed the mean at each site (Archer et al., 2017). Further, sponge-mediated DOC flux may vary as a direct function of the concentration of DOC in incurrent seawater, suggesting that there may be a threshold ambient DOC concentration above which sponges switch from net production to net consumption of DOC (Mueller et al., 2014; McMurray et al., 2016; McMurray, Pawlik & Finelli, 2017; Archer et al., 2017; Morganti et al., 2017). Given the putative relationship between ambient DOC availability and DOC flux, Morganti et al. (2017) were unable to conclude that the lack of DOC uptake observed for the sponge *Crambe crambe* was due to low concentrations of ambient DOC during their experiment rather than the inability of the sponge to process DOC; indeed, this threshold relationship for the net uptake of DOC may render moot any inter- or intraspecific comparisons of DOC flux (Table 1), and future work on sponge feeding should include DOC concentration as a covariate.

Another factor that likely confounds rate estimates of sponge feeding is the heterogeneous composition and nutritional value of food resources available to sponges (i.e., DOC, LPOC, detritus). Sponges are generally thought to feed on the labile, rather than refractory, fraction of DOC (Yahel et al., 2003; de Goeij et al., 2008b). Recent reports suggest that sponges consume algal-derived DOC at higher rates than coral-derived DOC (Rix et al., 2017). Additionally, while both symbiotic microbes and sponge cells have been implicated in DOC uptake (de Goeij et al., 2008a; Rix et al., 2016, 2017), relative rates of DOC uptake by microbes vs. sponge cells may differ (Rix et al., 2017). Moreover, there is growing evidence that sponges are not indiscriminant suspension feeders, but rather actively and selectively feed on available planktonic foods (Maldonado, Ribes & van Duyl, 2012). Sponges preferentially consume POC relative to DOC, perhaps due to the relatively lower C:N ratio of the former, and diet selection may enable sponges to increase nutritional gains (McMurray et al., 2016), further challenging the idea that the relative availability of living picoplankton based on measurements from episodic sampling schemes is a valid proxy for food limitation of sponges.

Detritus can also constitute a significant portion of the sponge diet; although, relative to DOC, there are far fewer quantitative studies of sponge-mediated detrital flux. Detritus was found to make up approximately 20% of the TOC and 54% of the POC consumed by the sponges *X. muta* (McMurray et al., 2016) and *Negombata magnifica* (Hadas, Shpigel & Ilan, 2009), respectively. For other species, detritus consumption appears to be negligible (Ribes, Coma & Gili, 1999a; Yahel et al., 2003). Similar to DOC consumption, estimates of detritus consumption by sponges are variable, likely due to the heterogeneous composition of the detrital pool, and estimates of sponge-mediated detrital flux can be confounded by the release of detritus. Further, in direct contrast to expectations for animals that are food-limited, there is a growing number of both HMA and LMA encrusting sponge species that contribute to the sponge-loop and are net producers of detritus (de Goeij et al., 2013; Rix et al., 2016, 2017). This detritus production is thought to result from the rapid proliferation and shedding of cells, primarily choanocytes, and is fueled by DOC consumption (de Goeij et al., 2009, 2013; Alexander et al., 2014, 2015). In fact, only 39–45% of the organic carbon consumed by the sponge *Halisarca caerulea* was used for respiration, with the remaining 55–61% of TOC uptake allocated to rapid cell turn-over and shedding (de Goeij et al., 2008b). For the giant barrel sponge *X. muta*, which is a net consumer of detritus (McMurray et al., 2016), DOC consumption alone accounts for 60–100% of respiratory demands, with the balance of organic carbon acquired likely allocated to sponge growth (Hoer et al., 2018). Similar to nearly all animals, sponges would be expected to assimilate more carbon provided that more is available; yet, this response does not directly imply the existence of food limitation. As indicated previously, it is likely that all organisms are resource-limited at some level, in that the provision of additional food or nutrients at the right time in their life-cycle could result in incrementally greater growth or reproduction, but this individual-level response is not necessarily important at ecologically relevant scales of time or space (Pawlik et al., 2015a).

**Figure 1:**
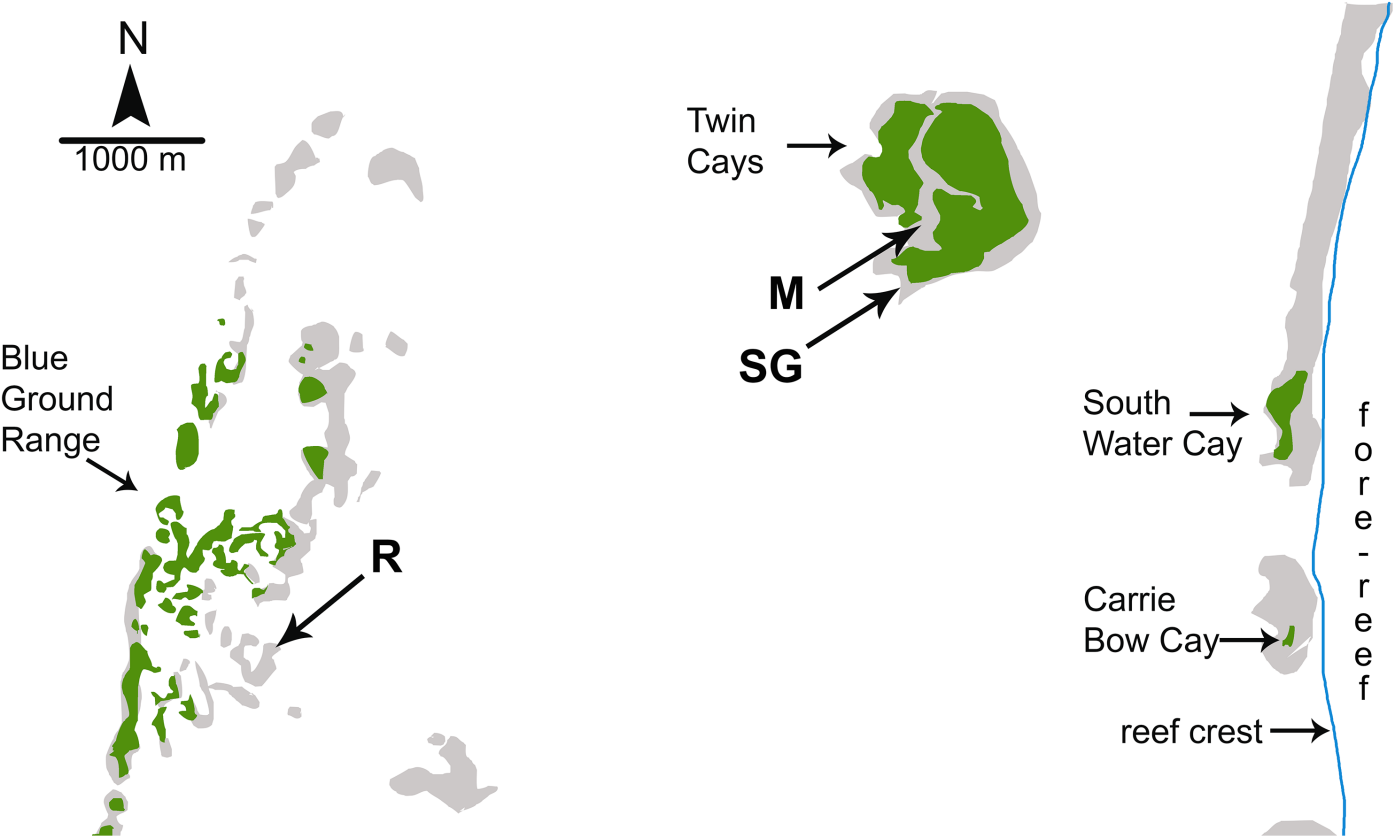
Map of study sites used by Wulff (2017). Map of study sites used by Wulff (2017) from Fig. 1.2 of Strimaitis (2012) and Google Earth satellite images. Reef crest of the Belizean Mesoamerican barrier reef is to the right. Emergent land (South Water Cay, Carrie Bow Cay) and mangroves (Twin Cays, Blue Ground Range) are shown in green, shallows and hard-bottom in light gray, reef crest is a line in blue. Extensive seagrass beds throughout lagoon area not shown. Experimental sites used by Wulff (2017) are mangrove (M), seagrass meadow (SG) and coral reef (R). Site locations are approximate based on available information. Site R lies ∼11 km due East from the outflow of the Sittee River.

Investigations of sponge feeding that have played a prominent role in the debate over the importance of bottom-up control of sponges on coral reefs were conducted in fore-reef environments where consistent water quality and the depth of the water column mitigate the biotic and abiotic variables associated with shallow-water environments; for example, foundational manipulative experiments were conducted on fore-reefs in the Florida Keys at depths of 12–30 m (Trussell et al., 2006; Pawlik et al., 2013). In a recent study designed to address both bottom-up and top-down controls on sponge growth, Wulff (2017) conducted reciprocal transplant experiments among three habitats that experienced different levels of food and predation: coral reef, mangrove, and seagrass meadow. However, all three of the experimental habitats used by Wulff (2017) were located inside the shallow back-reef lagoon between the Belizean continental coastline and the Mesoamerican barrier reef (Fig. 1). The habitat site designated as “coral reef” in the Blue Ground Range is closer to the Belizean mainland than the mangrove and seagrass meadow habitat sites and closer to the mouth of the Sittee River, with a depth easily accessible by snorkeling (<3 m). Most of the emergent Blue Ground Range is covered with mangroves (as can be seen using the satellite function in Google Earth), and references to this location comment on a bottom of “burrowed mud” and that the water is “usually turbid” (Rützler et al., 2000). Indeed, while the three sites in Wulff (2017) may have been different in terms of localized bottom cover, all three are lagoonal habitats strongly influenced by terrestrial run-off because of their proximity to the mainland and their very shallow depth. Wulff (2013) documented sponge mortality events at the Blue Ground Range site in 2007–2008 and again in 2010–2011 that resulted in the death of 49% and 71% of the sponge biomass, respectively, with substantial losses of the same species employed in the experiments she reports from one year before the die-off began, and attributed the more recent event to phytoplankton blooms. While use of the term “coral reef” is often debated by reef ecologists based on substratum formation and coral cover, past references to the ecology of sponges on Caribbean reefs have referred to the habitat seaward of the reef crest, in the fore-reef zone (Fig. 1), a habitat decidedly different from the mangrove and seagrass dominated back-reef lagoon in terms of depth, abiotic effects, flow regime and water quality (Nagelkerken et al., 2008). Indeed, the “polarized debate” over bottom-up and top-down control referenced by Wulff (2017) specifically refers to the fore-reef from ∼10 m to mesophotic depths (see Fig. 4 in Pawlik et al., 2015a; title of Slattery & Lesser, 2015), not back-reef lagoonal habitats.

In assessing the bottom-up component of between-habitat comparisons, Wulff (2017) analyzed picoplankton and nutrients from lagoonal water samples taken at <3 m depth in December 2009 and May 2010, without referencing the lagoonal phytoplankton blooms reported by Wulff (2013) for 2010–2011. Data from these water samples were reported as mean values of the total picoplankton concentrations (as cells/mL), and total dissolved nitrogen and DOC (as μM). On the basis of this limited water sampling scheme, Wulff (2017) concluded that the primary control of sponge growth on Caribbean coral reefs was bottom-up, because both sponge growth and picoplankton abundance were greater at mangrove vs. reef sites within the lagoon. As discussed above, picoplankton abundance alone is an inappropriate metric of food availability given the heterogeneous composition and nutritional value of the picoplankton pool and preferential feeding by sponges on select picoplankton types. Regardless, total mean picoplankton carbon available to sponges was actually greater at the reef site relative to the mangrove site in December 2009 (Strimaitis, 2012, Fig. 1.8), contrary to the statement in Wulff (2017) that there was a general pattern of greater food availability at the mangrove site that resulted in higher sponge growth. Mean picoplankton nitrogen was generally greater at the mangrove site over both days sampled (Strimaitis, 2012, Fig. 1.9), but this result may be confounded by the high C:N ratio conversion factor (20) used for the picoeukaryotic cell type (Strimaitis, 2012, Table 2.1). Total dissolved nitrogen was similarly higher at the mangrove site (Wulff, 2017), but the assumption that this is an indication of higher quality food is erroneous because the dissolved nitrogen pool includes both organic and inorganic forms, and sponges are known to be among the largest benthic sources of dissolved nitrogenous waste (Southwell et al., 2008). Further, Wulff (2017) did not consider (1) detritus, which can constitute a large portion of the diet for some sponge species (Hadas, Shpigel & Ilan, 2009; McMurray et al., 2016), (2) sponge feeding selectivity and foraging efficiency, which has been demonstrated to change as a function of food abundance and composition (McMurray et al., 2016), or (3) differences in DOC composition and nutritional value between habitats, which can dramatically affect sponge survivorship (Hunting et al., 2013b) and carbon uptake (Rix et al., 2016). Finally, the conclusion in Wulff (2017) of a bottom-up effect is undercut by the higher growth rate for sponges at the seagrass site relative to the reef site, despite ∼3-fold higher levels of carbon and nitrogen recorded at reef sites in December 2009 and similar levels between sites in May 2010 (Strimaitis, 2012, Fig. 1.8). In short, methodological problems associated with the study in Wulff (2017) preclude a greater understanding of bottom-up effects on Caribbean reef sponges, primarily because the experiments reported therein were performed in shallow lagoonal habitats where water quality is decidedly different from the fore-reef, and because the study lacked sufficient data on food availability to draw any conclusions about food limitation.

## Top-down Control—What’s Old?

Studies of ecology are complicated by the large number of biotic and abiotic factors that influence the distributions and abundances of organisms. Pattern and process is best revealed by choosing experimental conditions that limit complexity. As discussed above, in studies of coral reef ecology, research conducted on fore-reefs limit the primarily abiotic complexity associated with shallow, lagoonal and mangrove environments (Pawlik, McMurray & Henkel, 2007; Nagelkerken et al., 2008; Hunting et al., 2013a). For sponges on Caribbean reefs, properly controlled and measured manipulative experiments on fore-reefs have demonstrated that predatory fishes rapidly consume preferred sponge species, then move on to feed on chemically undefended, palatable sponge species, but avoid chemically defended species (Dunlap & Pawlik, 1996, 1998; Pawlik, 1997, 1998, 2011; Hill, 1998; Hill & Hill, 2002; Loh & Pawlik, 2009; Leong & Pawlik, 2010; Pawlik et al., 2013). These patterns have been validated by surveying fore-reefs across the Caribbean; again limiting complexity by specifically choosing sites on opposite sides of a spectrum: where sponge-eating fishes have been removed by decades of overfishing with fish-traps vs. sites where sponge-eating fishes have been protected from fishing by virtue of their remote location or marine protected area status (Loh & Pawlik, 2014; Loh et al., 2015).

While the studies listed in the previous paragraph have found evidence of top-down control of sponge communities by fishes on contemporary Caribbean reefs, this degree of top-down control likely pales in comparison to what Caribbean reef communities experienced historically, before hawksbill turtles (*Eretmochelys imbricata*) were reduced to less than 1% of their former population (McClenachan, Jackson & Newman, 2006). Hawksbill turtles feed almost exclusively on sponges (Meylan, 1988), and like sponge-eating fishes, prefer chemically undefended sponge species. As with studies of the feeding effects of fishes on sponges, the only appropriate method to assess predatory impact are gut content analyses, which are difficult to obtain for a rare and protected marine reptile. Table 2 lists the ranked abundance of sponge species found in the gut contents of hawksbill turtles from the seven studies that report this information. The sponge species listed in Table 2 are identified by chemical defense category from Loh & Pawlik (2014), and the relative proportion of palatable and chemically defended species are shown in the central columns of the table. While the metrics used to report sponge abundance in the guts of hawksbills are variable among studies, the preponderance of palatable sponge species in their diet is striking, with a particular preference across studies for *Chondrilla caribensis*, *Geodia gibberosa* and *G. neptuni* (Meylan, 1988; Alvarez, 2000; Stringell et al., 2016).

**Table 2:** Food preferences of Caribbean hawksbill turtles.

Ranked abundance	**Palatable**	Defended	Unknown	Citation
***Chondrilla caribensis*** (most abundant) *Ancorina* sp. ***Geodia*** sp. ***Placospongia*** sp. *Suberites* sp. ***Myriastra*** sp. *Ecionemia* sp. ***Chondrosia*** sp. *Aaptos* sp. *Tethya* cf. *actinia* (least abundant)	5 sp.	1 sp.	4 sp.	Meylan (1988) Ranked by product of average % dry mass and frequency of occurrence *n* = 54 turtles
***Geodia neptuni*** (48.2%) ***Polymastia tenax*** (30.4%) *Stellettinopsis megastylifera* (11.9%) *Coelosphaera raphidifera* (11.1%) ***Cinachyrella kuekenthalli*** (8.3%) ***Chondrilla caribensis*** (7.3%) ***Myriastra kalitetilla*** (7.4%)	81.5%	0%	18.5%	van Dam & Diez (1997) % occurrence in samples *n* = 110 lavage samples from at least 75 turtles
*Tethya* sp. (only one listed)			1 sp.	Mayor, Phillips & Hillis-Star (1998) *n* = 18 turtles
***Chondrilla caribensis*** (26.8%) ***Chondrosia collectrix*** (16.0%) ***Geodia gibberosa*** (12.4%) ***Geodia*** sp. (6.5%) *Erylus ministrongylus* (5.1%) ***Iotrochota birotulata*** (1.2%)	92.5%	7.5%	0.0%	Alvarez (2000) average % occurrence *n* = 146 turtles
***Chondrilla caribensis*** (22.4 ml) ***Myriastra kalitetilla*** (2.4 ml) *Tethya crypta* (0.8 ml) ***Spirastrella coccinea*** (0.7 ml) ***Geodia neptuni*** (0.6 ml)	97.0%	0.0%	3.0%	León & Bjorndal (2002) total volume (ml) *n* = 48 lavage samples
*Melophlus ruber* (75.3%) ***Chondrilla caribensis*** (14.1%) ***Geodia gibberosa/neptuni*** (5.4%) ***Cinachyrella*** spp. (4.9%)	24.5%	0.0%	75.5%	Berube et al. (2012) % composition by dry weight *n* = 5 turtles
***Chondrilla caribensis*** (27%) ***Geodia neptuni*** (17%) ***Halichondria melanadocia*** (16%) *Scopalina ruetzleri* (8%) ***Cinachyrella alloclada*** (5%) *Erylus formosus* (4%)	84.4%	15.6%	0.0%	Stringell et al. (2016) % wet biomass *n* = 45 turtles

**Note:**

Ranked abundance of sponge species from gut contents of hawksbill turtles identified by chemical defense category (Loh & Pawlik, 2014). Sponge species are coded by defense category as palatable (bold), defended (underlined) or unknown. Only species that constituted >1% of abundance metric used by the cited study are listed. Abundance metric used in cited study is indicated after species name. Relative percentage of palatable, defended and unknown species from the list is shown in the central three columns.

**Figure 2:**
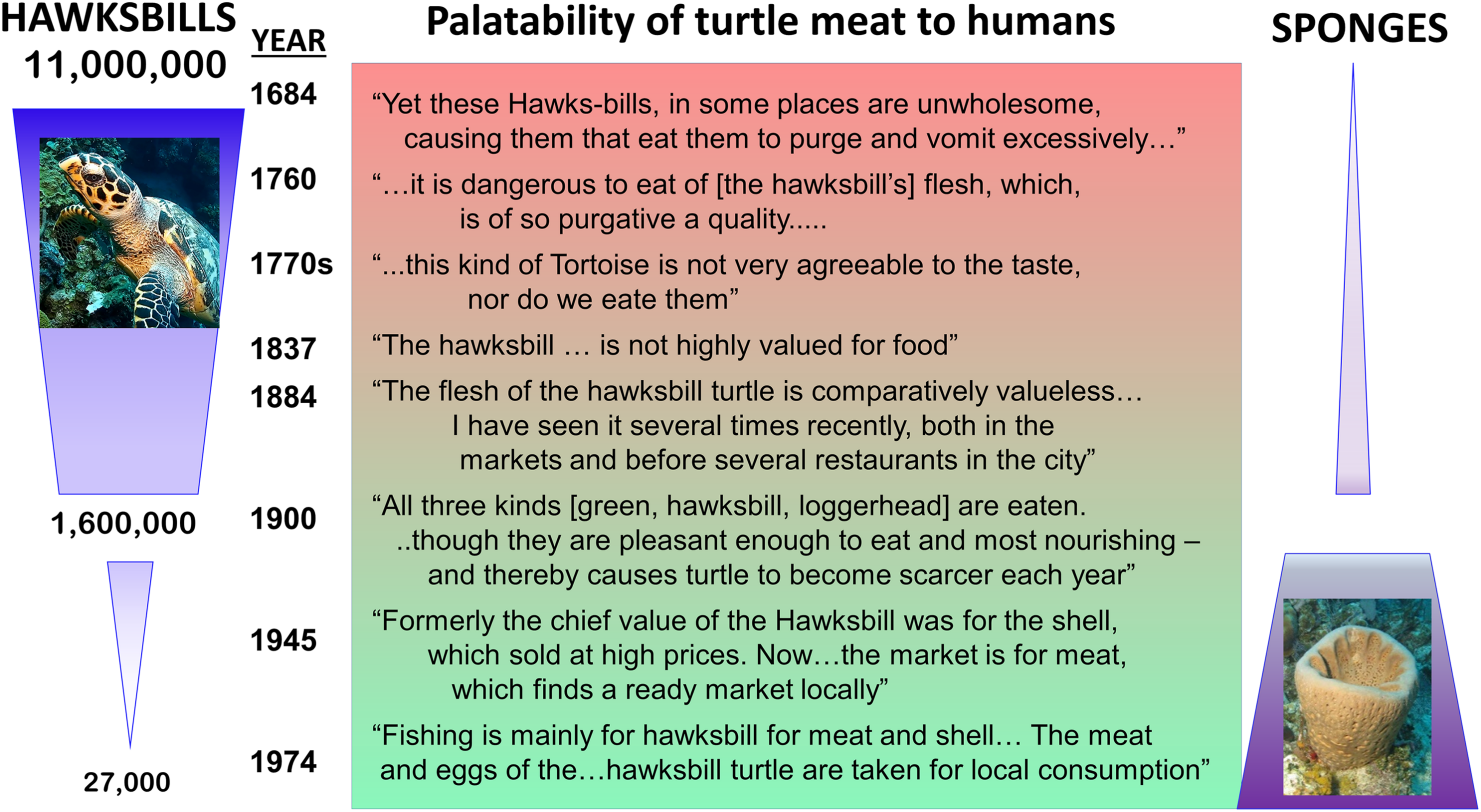
Top-down control of sponges on Caribbean reefs in the past. Relationship between estimated number of hawksbill turtles in the Caribbean region over time (left) and palatability of turtle meat to humans (center), based on ship captain’s logs (data from McClenachan, Jackson & Newman (2006)). Relative palatability of turtle meat suggests that historically large numbers of hawksbills were forced to graze chemically defended sponge species, rendering their meat distasteful to humans, while small numbers of turtles on contemporary reefs eat primarily undefended sponge species (right), and their meat is palatable to humans. Photographs by J.R. Pawlik.

**Figure 3:**
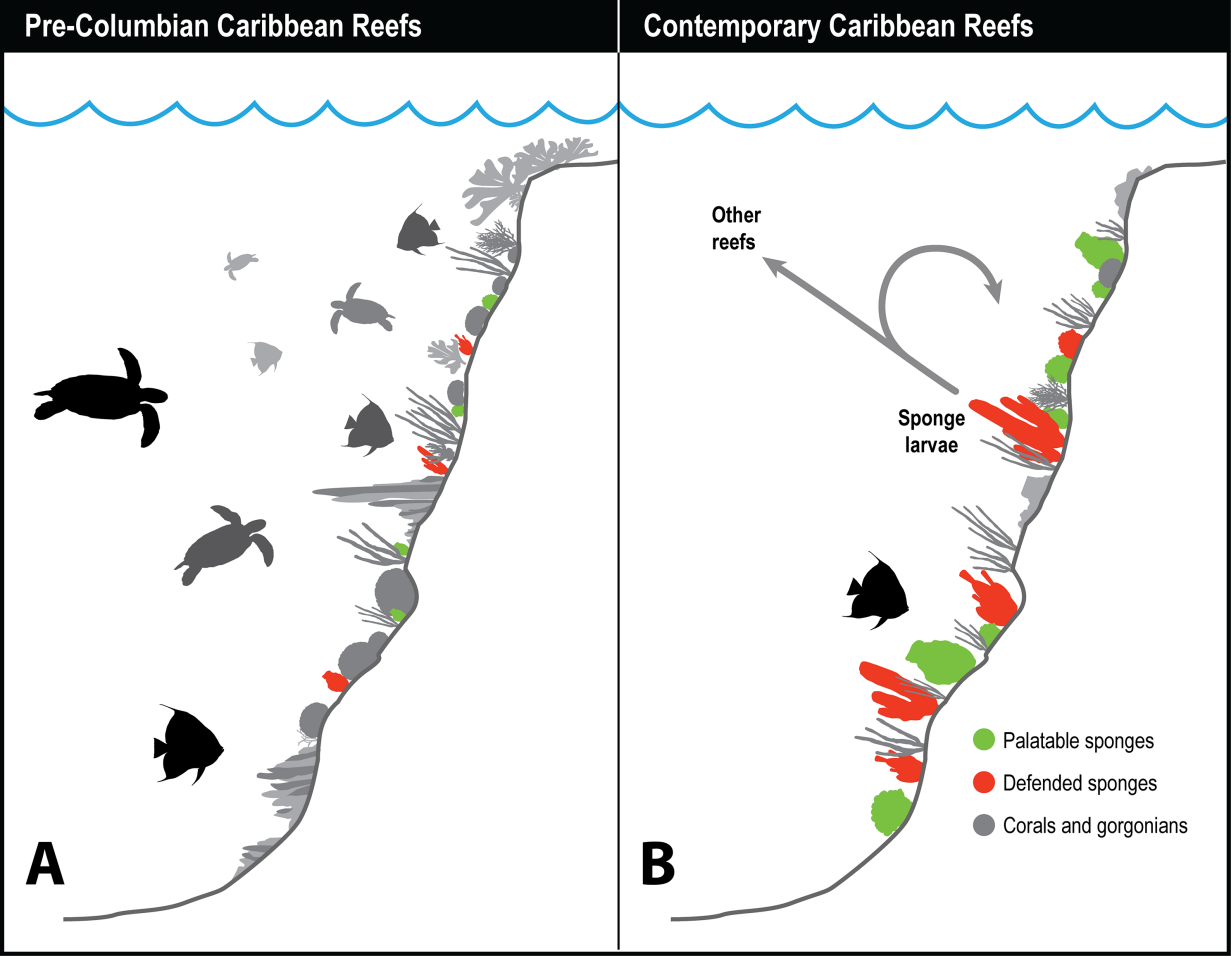
History of sponge communities on Caribbean fore-reefs. Comparison of likely impact of predators on sponge abundance on pre-Columbian and contemporary Caribbean reefs. (A) Large numbers of sponge predators, particularly hawksbill turtles, on pre-Columbian reefs kept sponges at low abundances, with both chemically defended and palatable sponge species relegated to refuge habitats. (B) Predator release on contemporary Caribbean reefs has allowed sponges to increase in abundance, with faster growing and reproducing palatable sponges dominating reefs where sponge-eating fishes have been removed by overfishing. Higher sponge biomass on contemporary reefs reinforces sponge dominance through recruitment, both to the same reef, and across reefs with different levels of fishing protection.

Historical records of the logs of ships’ captains and other sources suggest that hawksbill turtle flesh was toxic to humans before 1900 when these turtles were abundant, but that hawksbills became a source of food for humans after they became rare (Fig. 2), indicating that the estimated 11 million hawksbill turtles on pre-Columbian Caribbean reefs effectively grazed away the undefended sponge species and turned to eating chemically defended species that rendered their own flesh toxic to humans (McClenachan, Jackson & Newman, 2006). Therefore, while studies have concluded that there is top-down control of sponge communities on contemporary Caribbean reefs in locations where sponge-eating fishes are abundant compared to where they are scarce due to overfishing (Loh & Pawlik, 2014), top-down control was decidedly more intense in the past, when both sponge-eating fishes and hawksbill turtles were grazing sponges from the reefs (McClenachan, Jackson & Newman, 2006). Juvenile hawksbills have a mean diurnal dive depth of 8 m, but with a range that exceeds 90 m and that increases with turtle size, indicating that turtle spongivory can extend well into the mesophotic zone (Blumenthal et al., 2009). It is likely that pre-Columbian reefs were stripped of emergent palatable sponge species as well as some defended sponge species by the combined grazing activities of much larger populations (and larger individuals) of both hawksbills and sponge-eating fishes, with some vertical partitioning of feeding habitat between turtles of different size classes based on their maximum dive depths (Fig. 3). Under these conditions, sponge species in all three defense categories (preferred, palatable, defended; Pawlik, 2011) likely only survived among the branches of hard corals, fire corals and chemically defended gorgonians where sponge predators were unable to access them, much as they currently do on contemporary reefs where sponge-eating fishes are abundant (Wooster, Marty & Pawlik, 2017). Indeed, the high diversity of sponges in the Caribbean, estimated to be in excess of 500 species (Miloslavich et al., 2010), is likely a consequence of the ability of sponges to survive as clonal animals of variable size and shape in refuge habitats ranging from great depths to the interstices of the reef crest, and including lagoonal habitats such as mangroves and seagrass beds.

Figure 3 summarizes the history of sponge abundance on Caribbean fore-reefs: by the middle decades of the 20th century, sponges began growing and recruiting outside of refuge habitats because the predatory activities of turtles and fishes had been greatly reduced on most reefs. Sponge colonization was tied to (1) reproductive output of larvae, which is a function of sponge tissue volume, and to (2) the availability of free space for recruitment. The precipitous loss of living coral cover in the 1980s and 1990s resulted in a large increase in free space for the recruitment of sponges and seaweeds on the dead coral surfaces, with seaweeds more rapidly colonizing the newly available substratum. In the decades that followed, sponge cover slowly increased, and is now on par with coral cover (Loh & Pawlik, 2014), although sponge biomass is likely orders of magnitude greater considering the relative difference in thickness of coral and sponge tissue. Further, sponges can survive in the dark interstices of reefs where light-dependent macroalgae and corals cannot (de Goeij et al., 2008b, 2013). The lack of resilience observed for Caribbean coral reefs relative to those in other parts of the tropics has been attributed (in its most simplified terms) to a feedback loop in which seaweeds provide DOC to feed sponges, and sponges provide inorganic nutrients to fertilize seaweeds (the “vicious circle,” Pawlik, Burkepile & Thurber, 2016).

Despite the foregoing experimental and correlative evidence of the impacts of predators on Caribbean fore-reef sponge communities, a recent paper reported that “top-down control was not detected within-habitat, on the coral reef” (Wulff, 2017, p. 1130). As previously indicated, this conclusion was based on sponge transplant and caging experiments conducted solely at <3 m depth in the lagoon between the Belizean barrier reef and mainland (Fig. 1). Wulff (2017) recorded no effect of predation on coral reef sponges in the lagoonal site designated as coral reef habitat, despite high abundances of sponge-eating fishes. These results are not surprising, however, because sponge-eating fishes preferentially feed on sponge species that are found abundantly in the seagrass and mangrove habitats that occur in the Blue Ground Range in close proximity to the “coral reef habitat” site used by Wulff (2017) (Fig. 1. The Blue Ground Range has a well described sponge fauna (Rützler et al., 2000), with species such as *Tedania ignis* and *Halichondria* sp. listed as “abundant;” these are the same sponge species that were rapidly devoured by angelfishes and parrotfishes when placed on the fore-reef in the Florida Keys at 15 m in video feeding experiments (Dunlap & Pawlik, 1996), and in subsequent predator-exclusion experiments (Pawlik, 1998), leading to their designation as “preferred” sponge species that could only persist in refuge habitats such as mangroves or seagrass beds, or in the interstices of the fore-reef (Pawlik, 1997; Fig. 7 in Pawlik, 2011). As in Wulff (2017), reef sponge species were ignored by sponge-eating fishes when placed next to preferred sponge species (*T. ignis* and *Halichondria* sp.) in feeding arrays on the fore-reef (Dunlap & Pawlik, 1996). The movement of reef fishes to mangrove habitats for the purpose of eating preferred sponge species not available on the fore-reef has been documented for fishes in lagoonal mangrove habitats in the Florida Keys (Dunlap & Pawlik, 1998), and the sponge-eating fishes surveyed by Wulff (2017) were likely similarly attracted to the mangrove and seagrass beds in the Blue Ground Range in the Belizean lagoon. As previously discussed for bottom-up effects, methodological problems associated with the study in Wulff (2017) preclude a greater understanding of top-down effects on Caribbean reef sponges because the study was conducted in a shallow lagoonal habitat where preferred sponge species associated with mangroves and seagrass beds were readily available to sponge-eating fishes.

Eleven years of replicated growth experiments conducted with many of the same reef sponge species used by Wulff (2017), but in fore-reef environments where preferred sponge species were absent, documented tissue loss due to grazing by sponge-eating fishes on faster-growing, chemically undefended sponge species, but not on slower-growing, chemically defended sponge species (Leong & Pawlik, 2010; Pawlik et al., 2013). Sponge growth experiments conducted in the Florida Keys (Leong & Pawlik, 2010; Pawlik et al., 2013) employed the more precise and accurate method of determining the wet mass of each sponge piece on an electronic balance at the beginning and end of the experiment, rather than a method of estimating volume from linear measurements of sponge pieces and deriving “conglomerations of appropriate geometric solids” (Wulff, 2017). Volume estimates from linear measurements are particularly problematical for small sponges, because the proportional error in visual estimates of volume increase with decreasing size. Wulff (2017) does not report the initial size of sponge transplant pieces, but indicates that the largest volume measurement for pieces of *Aplysina fulva* after four years was 388 cm^3^ and after nine years for *Ectyoplasia ferox* was 213 cm^3^. These volumes, as spheres, would have radii of 4.5 and 3.7 cm after four and nine years of growth, respectively, suggesting that initial sponge transplant pieces were less than the size of a tennis ball. The volume of a tennis ball with a diameter of 6.6 cm is 150.5 cm^3^, to which a +1 cm error in measurement yields a volume of 229.9 cm^3^, an increase in volume of 53%. Rather than simple spheres, living sponges have shapes that are asymmetrical and highly complex. It seems unlikely that precise and accurate linear measurements could be taken while an investigator was working underwater that would result in a volume determination from the summation of complex geometric solids. As discussed previously (Pawlik et al., 2015a), the only valid method for measuring the growth of small sponges over short periods of time is wet mass determination, which requires brief exposure of sponge pieces to air when weighing in a standardized manner. Sponge pieces experience 100% survival after this process when they are returned to a depth greater than ∼5 m, because any bubbles in their aquiferous system are reduced by pressure and expelled (Pawlik et al., 2013), but this technique would likely not have worked at lagoonal sites in <3 m of water depth.

**Figure 4:**
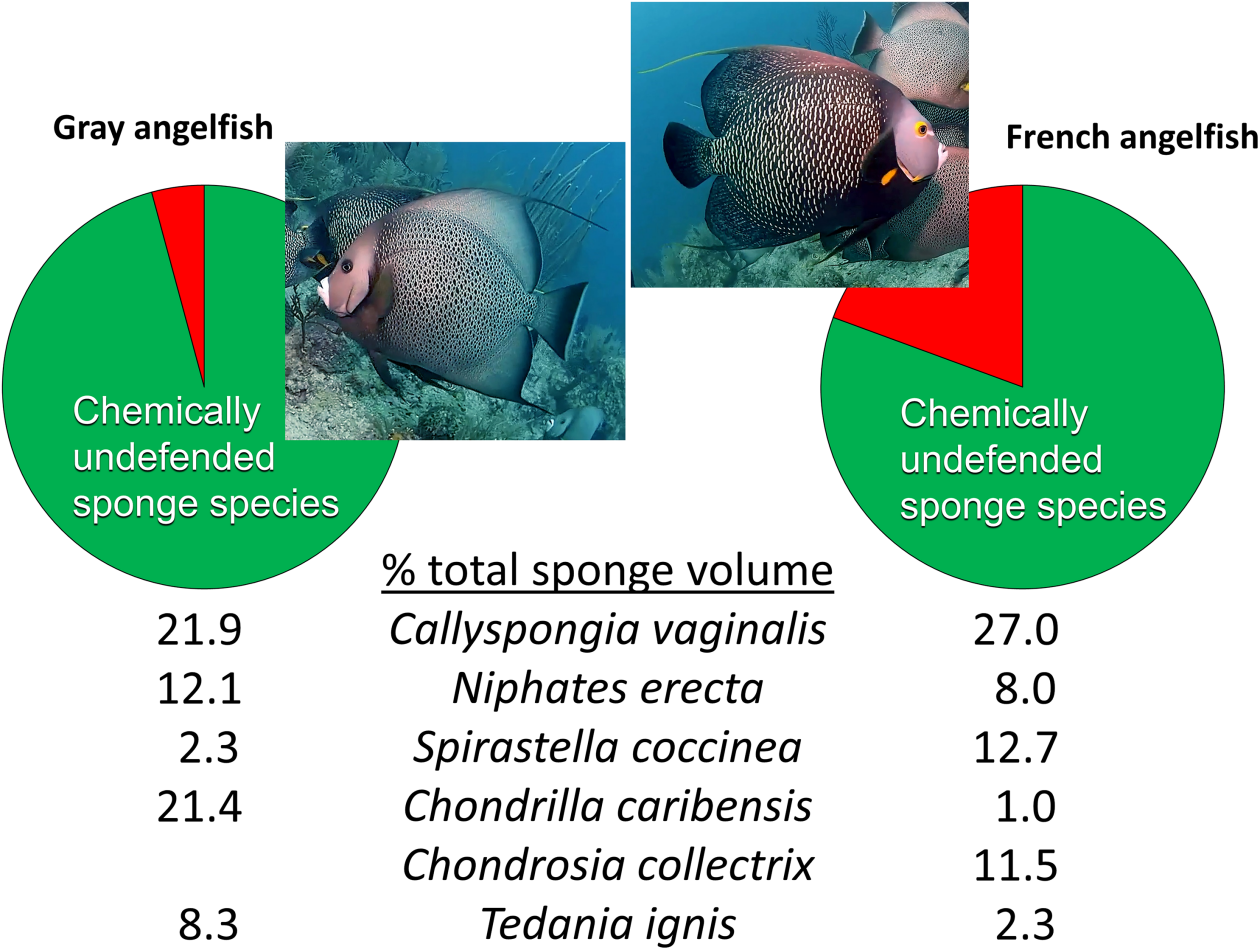
Food preferences of sponge-eating Caribbean angelfishes. Sponge tissue volume recorded from the guts of Gray and French angelfishes by Randall & Hartman (1968). Pie charts show the total proportion of identifiable sponge species that are in the chemically defended (red) and undefended or palatable (green) categories, based on bioassay data (Loh & Pawlik, 2014). Mean percentage of total sponge volume in the gut made up of the top six sponge species, all of which are in the palatable category, are shown for each fish species. Photographs by J.R. Pawlik.

Beyond reciprocal transplant experiments designed to test top-down effects through changes in sponge volume, an alternative methodology common in the literature is to observe the activities of predators in the field. The conclusions of Wulff (2017) were little changed from Wulff (1994), maintaining that sponge-eating fishes have little impact on sponges in reef habitats because of a smorgasbord feeding strategy: “Field observations of unmanipulated angelfishes feeding on live sponges have unambiguously confirmed that angelfish consume small amounts of many species in rotation” (Wulff, 2017, p. 1137). The citations used to support this statement, save one, are from field observations by divers or snorkelers of these fishes biting on different substrata with no direct measurements of the volume of sponge tissue consumed. As is the case for determining the diet of hawksbill turtles (see above), the technique of investigators following sponge-eating fishes in the field and observing biting behavior is an indirect and imprecise method for determining the volume of sponge tissue consumed, because (1) bites do not translate into tissue volume consumed, and (2) the short periods of observational time do not necessarily reflect the actual feeding behavior of these fishes, either because fishes are distracted by the observation process, or because feeding by fishes primarily occurs at times of the day when observations are less likely to occur (such as early morning or dusk). The remaining, methodologically valid, work cited by Wulff (2017) to support rotational feeding by sponge-eating fishes is Randall & Hartman (1968), a study in which the gut contents of 107 angelfishes were inspected after dissection and the relative volume and identity of sponge tissue in the diet determined. However, Randall & Hartman (1968) demonstrated that angelfishes have clear preferences in terms of the actual sponge tissue they ingest: the gray tube sponge *C. vaginalis* made up 22% and 27% of the sponge diet of the Gray and French angelfishes (*Pomacanthus arcuatus* and *Pomacanthus paru*), respectively, with four additional highest-ranked sponge species, all palatable species, making up 44% and 35% of the total volume, so that only five species of palatable sponges constituted 66% and 62% of the diet, respectively (Fig. 4) (Randall & Hartman, 1968). Indeed, several individuals of these two angelfish species can be seen repetitively biting, and removing tissue from, a naturally occurring (nontransplanted) specimen of *C. vaginalis* in a video taken at 15 m depth on Conch Reef, Florida Keys, on 1 July 2013 (Pawlik, 2013), corroborating the preference data of Randall & Hartman (1968) and clearly demonstrating repetitive and determined feeding rather than rotational or “smorgasbord” feeding.

It is interesting to note that Randall & Hartman (1968) assumed that the tissues of Caribbean sponges were broadly protected against fish predators, including species such as angelfishes that consumed them. Nevertheless, far from suggesting that sponge-eating fishes were rotational feeders, they acknowledged the preference for *C. vaginalis* and *C. caribensis* in particular, writing, “Thus it is probable that they are actively selected by their predators” (Randall & Hartman, 1968, p. 223). The gut content data of Randall & Hartman (1968), which constitute a study unlikely to be repeated anytime soon because of its reliance on the spearing and dissection of large numbers of reef fishes, could not be fully understood until laboratory and field studies revealed differences in the chemical defenses of sponge species (Chanas & Pawlik, 1995; Pawlik et al., 1995). When the gut content data were cross-referenced with chemical defense data, it was evident that sponge-eating fishes were focusing their attention on chemically undefended species, which constituted 96% and 81% of the identifiable sponge diet for Gray and French angelfishes, respectively (Fig. 4, Pawlik, 1997). Further, many of the sponge species listed by Randall & Hartman (1968) are not emergent on Caribbean fore-reefs, but are present in refuge habitats within reef interstices, suggesting that angelfishes search out and consume preferred sponge species that grow out of interstices, or are exposed when rubble is overturned by storms or by the feeding activities of larger vertebrates, such as turtles (Pawlik, 1998). As an example of this, Randall & Hartman (1968) found that the fire sponge, *T. ignis*, a species that is never observed as an emergent species on Caribbean fore-reefs, made up 8.3% and 2.3% of the sponge diet of Gray and French angelfishes, respectively (Fig. 4). When this preferred sponge species was removed from refuge habitats and placed on the reef in video experiments where the activities of the fishes could be tracked (Dunlap & Pawlik, 1996), and in caging experiments (Pawlik, 1998), it was rapidly consumed by repetitive biting, not only by angelfishes, but by parrotfishes. As another example, the orange icing sponge, *Mycale laevis*, can be found in a growth form that is tightly nestled in the interstices of living hard corals on reefs where sponge-eating fishes are abundant, but grows out of these cavities to form mounds that smother corals on reefs where sponge-eating fishes have been removed by overfishing (Loh & Pawlik, 2009; Loh et al., 2015).

## Summary and New Research Directions

As a hypothesis, exclusive bottom-up control of sponges on Caribbean fore-reefs was based on the sole reliance by sponges on living particulate food (Lesser, 2006; Trussell et al., 2006), when it had been known that sponges derive nutrition from DOC for over four decades (Reiswig, 1974). Recent studies have revealed that DOC and detritus play a much larger role in sponge nutrition than living particulate food (McMurray et al., 2016; Rix et al., 2016), and that there are abundant sources of labile DOC on Caribbean reefs (Wild et al., 2004; Haas et al., 2011, 2013). Where sponges have access to a substantial water column, such as on Caribbean fore-reefs, food limitation is unlikely to occur because of the wide variety of nutritional sources available to sponges, including particulate food, both living (microbes) and dead (detritus), dissolved food, both labile and refractory, and nutrition provided by symbiotic microbes, both heterotrophic and photosynthetic (Pawlik et al., 2015a; Pawlik, Burkepile & Thurber, 2016). Indeed, the most compelling argument against bottom-up control of sponges on Caribbean reefs is the high abundance, great variety of morphologies and co-occurrence of species in different microbial symbiont categories on Caribbean reefs compared with the oceanic Indo-Pacific reefs, where sponges are rare and mostly foliose phototrophic species (Pawlik et al., 2015a). Recent studies appear to be converging on the general understanding that most sponges on Caribbean fore-reefs rely on some combination of DOC, detritus, or symbionts for the carbohydrates that fuel their metabolic needs, and require living picoplankton for the other primary metabolites (amino acids, nucleic acids, lipids, etc.) that are used mainly for repair, growth and reproduction, or that some sponge species rely on microbial symbionts to provide primary metabolites that contain N and P. With the discovery that sponges are “optimal foragers,” that can change their food consumption choices depending on food availability (McMurray et al., 2016) has come the realization that sponges are able to integrate their nutritional uptake over time to take advantage of pulses of higher-quality foods and thereby avoid, not only food limitation, but nutrient (N, P) limitation.

Ripe for further research is the importance and contribution of DOC to sponge nutrition. DOC remains a “black-box” of presumed labile and refractory molecules dissolved in seawater (Nebbioso & Piccolo, 2013), part of the larger category of dissolved organic matter (DOM), which also includes dissolved organic nitrogen (DON) as a potential source of a limiting nutrient. New technologies are rapidly being brought to bear on the concentration, isolation (Li et al., 2017b) and identification of DOM constituents (Li et al., 2017a; Yuan et al., 2017) which should allow for the measurement of concentration changes for individual DOM compounds as they pass through sponges using In–Ex experiments (Fiore, Freeman & Kujawinski, 2017). Additionally, although mean values of sponge-mediated carbon and nutrient uptake are often used as the currency by which sponge feeding is reported and compared (e.g., Table 1), greater scrutiny of variation in sponge feeding may lead to an even greater understanding of sponge nutrition. Given recent findings that suggest that sponge and microbial cells may process different DOM types at different rates (Rix et al., 2017), there is a need to quantify further the relative contributions of these components from different members of the sponge holobiont to the overall rate of DOM uptake. Similarly, additional investigation is warranted to address the putative relationship between ambient DOC concentrations and DOC uptake. Indeed, a model similar to that established for planktonic microbes in the deep sea (Middelburg, 2015), may be helpful in elucidating sponge vs. microbial DOC uptake of different fractions of the DOC pool.

Where might food or nutrient limitation of sponges occur? We propose three sets of conditions under which food or nutrient limitation of sponges is likely:

1. **Seawater volumes are limited or stagnant and subject to re-processing and removal of available food by sponge pumping.** Water volume may limit sponge feeding in shallow-water lagoonal habitats, including mangroves and seagrass beds that experience periods of low flow. The food limitation effect in lagoonal habitats would be enhanced in places where sponge biomass is particularly high, such as in locations where mangrove prop roots support high sponge biomass (Pawlik, McMurray & Henkel, 2007; Wulff, 2017), or “sponge ground” habitats in places like Florida Bay where dense populations of large loggerhead sponges (*Speciospongia vesparium*) grow on hard-bottom in a few meters of water depth (Peterson et al., 2006). Cryptic reef interstices that experience low flow may also be food limited. This effect may significantly restrict the distribution and biomass of cryptic sponge species, limiting them to interstices in sections of the reef that are flushed by strong wave-induced surges (reef crests) or tidal flow (reef cuts). Estimates of carbon returned to reef environments through the sponge-loop may assume that cryptic interstices in the reef framework are habitat for encrusting sponges from the reef crest to great depths, but this has not yet been demonstrated.
2. **DOC is relatively unavailable, independent of POC.** It has been argued that sponges on Caribbean reefs live in a nutritionally richer environment (Wilkinson & Cheshire, 1990), with higher levels of labile DOC from the exuded photosynthate associated with high seaweed cover and higher levels of refractory DOC from terrestrial river inputs (Pawlik, Burkepile & Thurber, 2016). Conversely, oceanic reefs in other areas of the tropics are likely to fall on the opposite end of the spectrum in terms of DOC availability. The combination of low sponge cover and biomass, and the preponderance of phototrophic sponge species that are net producers of fixed carbon and grow in the foliose form of autotrophic, light-dependent corals has previously been described for these oligotrophic reefs (Wilkinson & Cheshire, 1990). Open-ocean reefs of the Indo-Pacific are bathed in seawater with comparable concentrations of living picoplankton, including heterotrophic bacteria, *Synechococcus* and *Prochlorococcus* (Haas et al., 2016). Yet, these locations may not have sufficient carbohydrate food value to sustain sponge metabolic needs, even if living POC in the water column is sufficient to sustain the nutrient requirements for sponge growth and reproduction.
3. **Living POC is insufficient, independent of DOC.** Sponges may be food-limited in the deep sea at depths below the mesophotic zone with reduced availability of nutrient-rich picoplankton. Food limitation may explain the faunal transition from demosponges to hexactinellid glass sponges along a vertical gradient of reef escarpments into the deep sea. Water column DOC in the deep sea is thought to be particularly refractory, having been subjected to re-processing by microbial metabolic activity over a long period of time (Hansell, 2013). Interestingly, studies of deep sea glass sponge reefs at higher latitudes indicate that cold-water hexactinellids do not consume DOC (Yahel et al., 2007), suggesting that their metabolism is adapted to consumption of the sources of living POC and detritus that remain available to sponges below the mesophotic zone.

The foregoing conditions under which sponges may be food-limited are found in habitats where comparative water sampling and analysis can be performed to test for differences in POC and DOC, including In–Ex experiments on sponges with the appropriate morphologies that distinctly separate incurrent from excurrent water flow through the sponge. Manipulative experiments or correlative studies to directly test differences in sponge growth among these habitats would be practicable in some cases (loggerhead sponges in lagoon habitats) but much more difficult in others (deep sea), because they would require sufficient replication, caging to exclude predators, and most importantly, appropriate measurement of sponge growth (wet mass determination of whole sponges rather than volume approximations based on linear measurements or the linear extension of sponge tubes; see Pawlik et al., 2015a).

Regarding top-down control, as indicated in a previous review (Pawlik et al., 2015a), the best controlled growth experiments performed to date to test the relative importance of top-down from bottom-up processes on sponge growth on coral reefs were done on the fore-reef at 15 and 30 m depth in the Florida Keys on Conch Reef, where patterns of particulate food availability have been established through repetitive seawater sampling for over two decades, and the initial and final mass of sponges were determined to provide accurate growth determinations (Pawlik et al., 2013). In addition to the two consecutive years of these experiments on Conch Reef, there were nine years of sponge growth experiments designed to test top-down effects on another fore-reef site, North Dry Rocks Reef (Leong & Pawlik, 2010). All of these exclusion growth experiments revealed strong effects of predation on chemically undefended sponge species, and these manipulative studies were subsequently validated by cross-Caribbean surveys of fore-reef sites that were intensively overfished or protected from fishing and had corresponding abundances of sponge-eating fishes (Loh & Pawlik, 2014).

Should the populations of hawksbill turtles continue to rebound, their impacts on sponge communities would be predictable, beginning with a reduction in palatable sponge species on Caribbean reefs (Table 2). Decades-old restrictions on turtle shell trade and fisheries designed to protect adult turtles, and more importantly, protection of turtle eggs on nesting beaches, have resulted in significant upward trends in hawksbill populations in the Caribbean (Mazaris et al., 2017). One recent intensive study of sea turtle abundance over a six-year period on Glover’s Reef Atoll, Belize, estimated 1,000–2,000 juvenile hawksbills in a ∼22 km^2^ fore-reef study area (Strindberg et al., 2016). Time-series data on the abundance of sponge species across the Caribbean would be a useful tool for assessing shifts in sponge community structure with changes in turtle populations. Interestingly, despite evidence that sea turtle populations in the Caribbean are increasing, growth rates of hawksbills have significantly declined since 1997 (Bjorndal et al., 2016), a trend that was linked to climate warming. While the reasons for this growth rate decline are mysterious and likely complex, one possible hypothesis is that increasing populations of juvenile hawksbills have already begun to deplete the most nutritious, accessible and palatable sponges on shallow fore-reefs, forcing turtles to expend more time and energy with less-preferred sponges and to dive deeper to find food, thereby reducing their rate of growth.

Largely unexplored is whether top-down control of sponge communities on Caribbean fore-reefs changes as a function of depth. One study has reported reduced levels of chemical defenses in a common sponge species across a depth gradient from shallow water to the mesophotic (Slattery et al., 2016), suggesting a concomitant change in predation pressure. Air-breathing hawksbill turtles are mostly restricted to shallow reefs (see above), and the abundance of angelfish species that prey on sponges was reported to be greatly reduced on vertical escarpments below 46 m at sites in the Bahamas and Cayman Islands (Slattery et al., 2016). Invertebrate predators of sponges, such as seastars, nudibranchs, and cowries (Pawlik & Deignan, 2015) may be more important at mesophotic depths where their own predators are less common. Indeed, Caribbean fore-reefs at the depths of the mesophotic zone and into the deep sea are ripe for study: how do changes in the diversity and biomass of demosponges and glass sponges track with sponge predators and sponge food sources as a function of depth? The answer to this question will likely also help to further our understanding of ecosystem dynamics on shallow reefs, both in the Caribbean and elsewhere in the tropics.

## Additional Information and Declarations

### Data Availability

The following information was supplied regarding data availability: The literature review did not generate any data or code.
